# Clinical Evaluation of Basal-Bolus Therapy Delivered by the V-Go^®^ Wearable Insulin Delivery Device in Patients with Type 2 Diabetes: A Retrospective Analysis

**DOI:** 10.3390/pharmacy8040215

**Published:** 2020-11-14

**Authors:** Trisha Zeidan, Carla Nikkel, Beth Dziengelewski, Stephanie Wu, Aleda M. H. Chen

**Affiliations:** 1 Bull Family Diabetes Center, Premier Health, Dayton, OH 45409, USA; tlzeidan@PremierHealth.com; 2 Zealand Pharma US Inc., Boston, MA 02210, USA; bdziengelewski@zealandpharma.com; 3 School of Pharmacy, Cedarville University, Cedarville, OH 45314, USA; stephaniewu@cedarville.edu (S.W.); amchen@cedarville.edu (A.M.H.C.)

**Keywords:** type 2 diabetes, V-Go, glycemic control, insulin, hemoglobin A1c

## Abstract

Insulin therapy is frequently required to achieve glycemic targets (A1c) in type 2 diabetes (T2D); however, clinicians and patients face barriers with the complexities of multiple daily injection regimens. Patch-like wearable insulin devices, such as V-Go, may simplify and optimize this complexity. This study evaluated the change in A1C and insulin total daily dose (TDD) in a suboptimally-controlled (not achieving A1C targets) T2D population after switching to V-Go. A retrospective chart analysis at a diabetes clinic was performed to evaluate change in A1c measurements from baseline (V-Go initiation) to end of study observation. Of the 139 patients enrolled, A1C significantly decreased from baseline (−1.5 ± 1.79%; *p* < 0.001). Patients prescribed insulin at baseline (*n* = 122) used significantly less insulin TDD (−8 u/day; *p* = 0.006). The percentage of patients meeting the target of A1C < 8% increased from 14% at baseline to 48% at study completion (*p* = 0.008). Patients prescribed a basal-bolus regimen prior to V-Go achieved an A1C reduction of 1.5 ± 2.0% (*p* < 0.0001) and experienced the greatest reduction in TDD (−24 u/day; *p* < 0.0001). Thus, patients switching to V-Go from a variety of therapies at baseline experienced reductions in A1C while using less insulin, with a reduction in clinically relevant hypoglycemia, indicating the potential benefit of V-Go in optimizing and simplifying T2D care.

## 1. Introduction

Diabetes is a chronic and complex disease that affects more than 34 million Americans [1]. Improving glycemic control has been shown to reduce diabetes-related complications; even a 1% reduction in hemoglobin A1C (A1C) has been associated with reductions in diabetes-related deaths, microvascular complications and myocardial infarction [2]. A1C is widely used to guide patient care decisions, as well as evaluate provider and healthcare system performance. The Healthcare Effectiveness Data and Information Set (HEDIS), generated by the National Committee for Quality Assurance (NCQA), is commonly used as a performance improvement tool and includes proportion of patients meeting A1C thresholds as a performance indicator for diabetes. The HEDIS reports poor glycemic control as A1C > 9%; and glycemic control as A1C < 8.0% and < 7.0% in select populations [3]. These target thresholds are consistent with the American Diabetes Association (ADA) recommendation that a target of A1C < 7% is appropriate for many patients. However, a target of <8% may be appropriate for patients with long-standing disease, multiple comorbidities or at greater risk for complications including hypoglycemia [4]. 

Management of patients with type 2 diabetes typically begins with the initiation and titration of non-insulin glucose-lowering medications (NIGLM) [5,6]. Due to the progressive nature of the disease, intensification of therapy over time is required, and many patients will eventually require and benefit from insulin to maintain or improve glycemic control [5,6]. Insulin therapy in patients with type 2 diabetes is often initiated with a single daily injection of basal insulin. However, the addition of prandial insulin boluses may eventually be necessary [6]. Multiple daily injections (MDIs) with a basal-bolus insulin regimen are associated with barriers for both clinicians and patients, impacting initiation and adherence to insulin regimens. Clinicians face challenges due to limited time available to manage and educate a patient on complex insulin regimens [7]. Thus, in many ambulatory care settings, pharmacists play a significant role in providing education and adjusting therapy regimens in collaboration with other healthcare professionals, such as nurse practitioners, physician assistants, physicians, and certified diabetes educators. Despite this careful management by the healthcare team, patient barriers include fear of needles, injection site pain, and managing the impact to daily living when multiple injections per day are required [8]. Additionally, many patients feel that there is a stigma associated with administering insulin in public, resulting in avoidance of insulin injections when eating away from home [8,9]. Over 70% of physicians report that patients do not adhere to the insulin regimen prescribed. This non-adherence can prevent a patient from meeting glycemic goals, thereby increasing the risk of serious complications [10]. Further, due to the integration of insulin, patients can also experience hypoglycemic events, which can lead to impaired cognitive function, seizures, comas, and mortality. Hypoglycemia is a primary concern for both patients and clinicians and can be a factor in delaying the start of insulin therapy [7,10]. According to the American Association of Clinical Endocrinologists (AACE) treatment recommendations, choice of insulin therapy should factor in ease of use, and the regimen should be as simple as possible [5]. Technology that simplifies treatment can improve patient compliance, leading to improved glycemic control, and also allow for better consistency in therapy which may optimize the balance between achievement of A1C targets and frequency of hypoglycemia. Advances in insulin delivery, such as patch-like basal-bolus insulin delivery devices, have recently been incorporated into the ADA standards of care as an alternative to insulin pen or syringe delivery [11] and can be a means to simplify insulin delivery and address patient barriers.

**Figure 1:**
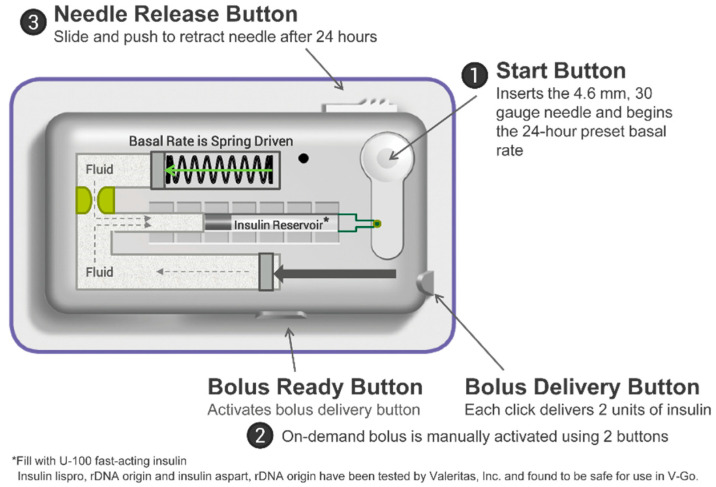
The V-Go Wearable Insulin Delivery Device.

V-Go (Zealand Pharma, Denmark) is a 24 h wearable patch-like insulin delivery device (Figure 1) that can deliver a continuous basal infusion (20, 30, or 40 units/24 h) and allows for up to 36 additional units of insulin for mealtime dosing in 2 unit increments [12].

V-Go has been cleared by the FDA for use in patients 21 years of age and older with a U-100 fast acting insulin. After filling the device from an insulin vial with the filling accessory provided, V-Go is affixed to the skin like a patch using a latex-free adhesive. Once placed, the patient presses the start button, which inserts a 4.6 mm, 30 gauge stainless steel needle into the subcutaneous tissue and initiates the delivery of the continuous basal rate of insulin over 24 h. On-demand bolus dosing at mealtimes in 2 unit increments is available by clicking two buttons consecutively. Bolus dosing can be done discreetly as the delivery button can be pressed through clothing. The V-Go device is fully disposable and is designed to be worn for 24 h before replacing with a newly filled V-Go.

Across multiple studies, use of V-Go has been associated with significant and clinically meaningful improvements in glycemic outcomes [13,14,15,16,17,18,19,20,21,22,23], and patients have reported high satisfaction with V-Go compared to prior insulin regimens [13,19]. As it is well understood that achieving optimized glycemic control can delay or defer macro- and microvascular complications, it is important to give patients tools to improve therapy adherence. Further, given the role of healthcare professionals, such as pharmacists, in managing diabetes collaboratively with patients, it is important to further elucidate whether switching to patch-like basal-bolus insulin delivery devices provides benefits to patients in real-world settings. Thus, the objective of this study was to evaluate the change in A1C and insulin total daily dose (TDD) in a suboptimally-controlled (not achieving A1C target of ≤ 7%) type 2 diabetes population after switching to V-Go.

## 2. Materials and Methods

### 2.1. Study Design and Criteria 

This study was conducted as a retrospective review of the electronic medical records (EMRs) at The Bull Family Diabetes Center in Dayton, Ohio, a comprehensive diabetes education and management outpatient clinic with care overseen by an Endocrinologist. Site standard practice is to offer insulin therapy with V-Go when insulin intensification or optimization is warranted due to suboptimal glycemic control. Patient education on use of the V-Go was provided by the site clinical staff, and written educational material and access to a customer care center were provided by the manufacturer, as is standard for all patients at this center using V-Go. Patients were managed per site standard of care, which included individualized treatment plans with shortened A1C testing intervals to evaluate the response to therapy for patients with uncontrolled diabetes, and less frequent A1C testing for patients with stable glycemic control. A systematic search of EMRs using keywords identified potential patients who were switched to V-Go from 1 January 2013 to 31 December 2016, and these patient records were evaluated for study eligibility. Inclusion criteria required (1) diagnosis of type 2 diabetes; (2) age ≥ 21 years; (3) baseline A1C > 7% and ≤14% within (−60/+7 days) of V-Go initiation; and (4) at least one documented follow-up A1C measurement within 6 months of V-Go initiation. Patients were excluded if prescribed U-500 insulin immediately preceding V-Go initiation or to fill V-Go, or if the patient utilized a traditional insulin pump immediately preceding V-Go initiation. For this study, V-Go was used as a device to administer a continuous basal infusion of insulin and on-demand bolus dosing over a 24 h period.

The primary endpoint was change in A1C from baseline to the final study visit collected. Secondary endpoints included change in insulin dose; percent of patients achieving an A1C < 8%; percent of patients achieving a reduction in A1C of ≥1%; change in A1C and insulin dosing based on baseline insulin regimen (basal-only, basal-bolus or premix regimens); changes in concomitant NIGLM; and hypoglycemic events (<70 mg/dL and <55 mg/dl). Patients served as their own control, as baseline characteristics were obtained. Wright State University Institutional Review Board reviewed and approved the study protocol and approved a waiver of informed consent. 

### 2.2. Data Collection

For subjects meeting the inclusion and exclusion criteria, an electronic data collection form was used to collect de-identified data for each patient including patient demographics, A1C, weight, body mass index (BMI), insulin doses and regimen, concomitant NIGLM, and documented patient-reported hypoglycemia. Data were extracted for the timeframe immediately preceding V-Go initiation through up to four follow-up visits on V-Go, where an A1C measurement was documented. Given the potential variability in timing of patient visits and in A1C testing intervals in this real-world study, data collection stopped upon reaching 4 visits, or subsequent to an A1C measurement documented after 6 months of intervention with V-Go. 

### 2.3. Data Analysis

Independent statistical analyses were performed by Cedarville University School of Pharmacy. Data were imported in SPSS v. 25.0 for analysis. Descriptive statistics (i.e., mean ± standard deviation, median, %, range) were used to report baseline variables. Paired t-tests or chi-squared tests, as appropriate, were used to determine differences from baseline for primary and secondary endpoints with a *p* < 0.05 denoting a statistically significant difference relative to the baseline data gathered. Change in A1C was based on the baseline A1C compared to the last documented A1C. To determine the impact of the change in A1C and change in TDD controlling for baseline A1C and overall change in NIGLM, a linear regression was performed. Change in NIGLM was included as a fixed effect.

## 3. Results

### 3.1. Study Population

A total of 154 patients were identified by the database query, of which 139 met study inclusion criteria. Leading reasons for exclusion were prior use of U-500 regular insulin; baseline A1C not in range or out of time window; no follow-up A1C available within 6 months; type 1 diabetes; and V-Go not initiated. Baseline characteristics including demographics and insulin regimens are shown in Table 1, and baseline concomitant NIGLM are shown in Table 2. Of the 139 patients enrolled, a majority (88%) were prescribed insulin prior to initiating V-Go, with over half of patients on a basal-only (53%) regimen (Table 1). The most commonly prescribed concomitant non-insulin medications were metformin, sulfonylurea, GLP-1 receptor agonists and DPP-4 inhibitors (Table 2).

### 3.2. Clinical Response with V-Go

**Figure 2:**
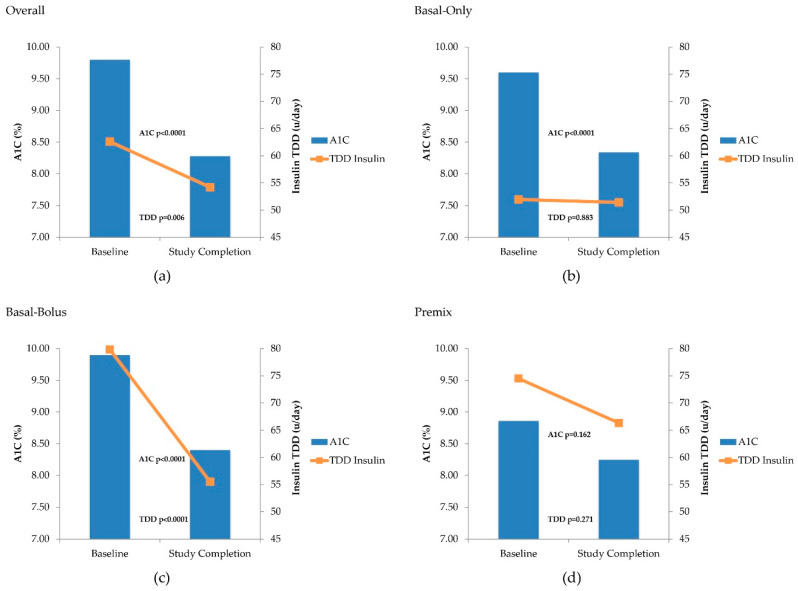
Change in A1C and Total Daily Dose (TDD) of Insulin with Use of V-Go. (**a**) Change in A1C for all patients (N = 139) and insulin TDD for all patients on insulin at baseline (*n* = 122); (**b**) change in A1C and TDD for patients on basal only at baseline (*n* = 73); (**c**) change in A1C and TDD for patients on basal-bolus at baseline (*n* = 36); (**d**) change in A1C and TDD for patients on premix at baseline (*n* = 13).

Figure 2 provides a visual overview of the changes in both A1C and TDD of insulin. In the graphic, the bars represent the A1C at baseline and study completion.

The scale for the A1C changes can be found on the primary y-axis (left-hand side of the chart). The lines represent the change in TDD of insulin (u/day), and the scale can be found on the second y-axis (right-hand side of the chart). For each, the *p*-value notes whether the change was significant. After a mean duration of 5 months between initiation of V-Go and the last patient visit recorded, the overall patient population experienced a statistically significant mean reduction in A1C (−1.5 ± 1.79; *p* < 0.0001) compared to baseline (Figure 2a). In addition, the percentage of patients achieving an A1C < 8% increased from 14% at baseline to 48% at study completion (*p* = 0.008), and the percentage of patients achieving an A1C ≤ 9% increased from 39% at baseline to 76% at study completion (*p* < 0.001). At completion, 58% of the population achieved a reduction in A1C of ≥ 1% with V-Go. Patients prescribed insulin at baseline (*n* = 122) experienced a statistically significant reduction in TDD of insulin (63 ± 39 u/day baseline, 54 ± 18 u/day study completion; *p* = 0.006) (Figure 2a). Normalized for weight, TDD of insulin was also statistically significant with a reduction from 0.62 ± 0.38 u/kg/day at baseline to 0.53 ± 0.17 u/kg/day at study completion (*p* = 0.002).

A significant reduction in A1C of 1.3 ± 2.0% from a baseline of 9.6% (*p* < 0.0001) was seen after initiation of V-Go in the cohort of patients using basal insulin only at baseline (*n* = 73), with no increase in TDD of insulin (52 u/day baseline to 51 u/day study completion) (Figure 2b). The cohort prescribed a basal-bolus regimen at baseline (*n* = 36) achieved a reduction in A1C of 1.5 ± 2.0%, (*p* < 0.001) from a baseline of 9.9%, using a lower TDD of insulin (80 u/day to 56 u/day, *p* < 0.001) after switching to V-Go (Figure 2c). Basal insulin TDD in this cohort was reduced from 48 u/day to 29 u/day (*p* < 0.0001). The cohort prescribed premix insulin at baseline (*n* = 13) achieved a non-significant reduction in A1C of 0.6 ± 1.5% from a baseline of 8.9%, with a non-significant reduction in TDD of insulin (74 u/day to 66 u/day). In the cohort of patients naïve to insulin at baseline (*n* = 17), a mean reduction in A1C of 3.3 ± 1.3% from a baseline of 11.1% (*p* < 0.001) was seen after initiating insulin therapy with V-Go. 

The number of patients using at least 1 NIGLM decreased by 4% from baseline to study completion, and the overall number of NIGLM prescribed decreased by 11% with V-Go compared to baseline, with reductions across all classes except metformin/sulfonylurea combinations, SGLT-2 inhibitors, and thiazolidinediones (Table 2). In the regression analysis, change in NIGLM did not significantly impact the change in A1C or TDD. 

### 3.3. Safety Assessments

While not significant, the overall percentage of patients reporting hypoglycemia (<70 mg/dl) at the baseline visit versus at the study completion visit decreased after initiating V-Go, with a 45% decrease in patients reporting clinically relevant hypoglycemia (<55 mg/dl) (Table 3).

Across baseline insulin regimen subsets, reported hypoglycemia (<70 mg/dl) at baseline vs. at study completion, respectively, was 23% vs. 19% for the basal only cohort; 46% vs. 33% for the basal-bolus cohort; and 38% vs. 23% for the premix cohort.

A mean increase in weight from 102.4 ± 19.0 to 103.3 ± 19.3 kg (*p* = 0.011) was observed from baseline to study completion in the overall patient population; however, there was no significant increase in weight for the cohort of patients previously prescribed insulin (102.1 kg baseline, 102.8 kg study completion; *p* = 0.074).

## 4. Discussion

Performance measures and quality improvement have been a focus of diabetes care for many years to improve care and reduce complications, and pharmacists play a significant role in ensuring patients meet these goals. Improvements in glycemic control, even when moderate, can reduce the risk of complications and optimize patient outcomes. An expert panel convened by the ADA in 2011 recommended using performance measures that account for multiple factors: an individual patient’s risk status; relative benefits of treatment options; and patient preferences [24]. In addition, the ADA suggests less stringent glycemic goals (A1C < 8%) are appropriate in patients with advanced or long-standing disease, at risk for hypoglycemia, and in those preferring a less burdensome therapy [25].

This retrospective analysis of 139 patients with type 2 diabetes switched from prior anti-hyperglycemic therapy to basal-bolus insulin therapy with V-Go showed a significant and clinically relevant 1.52% reduction in A1C using a lower TDD of insulin after a mean of 5 months of therapy. These findings are noteworthy, considering this complex population had a mean baseline A1C of 9.8%, mean duration of diabetes over 13 years, and nearly 90% of these patients with sub-optimally controlled diabetes were using insulin at baseline. Poor glycemic control has been associated with microvascular and cardiac complications, and even a 1% decrease in A1C reduces the risk of these complications [2]. In the current study, 58% of patients achieved a reduction > 1% in A1C after a mean duration of 5 months with V-Go; the proportion of patients achieving an A1C < 8.0% more than tripled; and the proportion of patients with poor glycemic control (A1C > 9%) was reduced by 60%. Concomitant NIGLM were recorded and compared at baseline and study completion; the proportion of patients using at least 1 NIGLM as well as the overall number of NIGLM prescribed both decreased from baseline to study completion. The improvements in glycemic control achieved with V-Go were attained using less TDD of insulin as well as fewer NIGLM. The improvement in glycemic control reported in this study is consistent with previous V-Go research. A study conducted by Sutton et al. showed a significant reduction in A1C of 1.79% (*p* = 0.002) after transitioning to V-Go, with 50% of patients achieving an A1C < 8% compared to only 17% prior to V-Go therapy (*p* < 0.001) [21]. 

Despite improvements in insulin delivery such as insulin pens, adherence to basal-bolus insulin therapy has been challenging due to the complexities and burdens involved with the multiple injections required. The basal-bolus cohort in the current study gives insight into the impact of insulin delivery method on glycemic control. Patients using a basal-bolus MDI regimen prior to initiating V-Go experienced a significant decrease in A1C of 1.5% and achieved this improvement in glycemic control using 30% less insulin. In this cohort, both basal TDD and prandial TDD were decreased with use of V-Go. Previous research in patients using a basal-bolus regimen prior to switching to V-Go supports the improvement in glycemic control in this population. In the study conducted by Sutton et al., the cohort of patients using a basal-bolus MDI regimen prior to switching to V-Go achieved a 1.5% reduction in A1C (*p* < 0.001) using 31 u/day less insulin TDD (*p* < 0.001) after 6 months of V-Go use [21]. 

A prospective study conducted by Cziraky et al. randomized 52 study sites via cluster randomization to V-Go or to standard treatment optimization (STO) and showed a greater decrease in A1C with V-Go than STO (V-Go −1.0%; STO −0.5%; *p* = 0.002) after 4 months. There was a significant decrease in TDD of insulin with V-Go (0.76 u/kg to 0.57 u/kg; *p* < 0.001), but no change in insulin use with STO (0.72 u/kg). Over half of the patients enrolled used a basal-bolus MDI regimen prior to switching to V-Go. This cohort experienced a significantly greater A1C reduction with V-Go compared to the STO group (−1.0% vs. −0.4%, respectively; *p* = 0.006), and V-Go patients required significantly less insulin than the STO group [14].

Method of delivery of basal-bolus therapy matters; data support improvement in glycemic control using less insulin when switching from MDIs to V-Go. Patch-like wearable basal-bolus insulin delivery devices such as V-Go are included in the 2019 Standards of Medical Care in Diabetes issued by the American Diabetes Association [11], and real-world evidence including the current study demonstrates the improvements in glycemic control that may be achieved with V-Go. V-Go removes the complexities associated with multiple daily injections; thereby it may simplify education for the care team and patient, minimizing the burden to patients, and allowing for discreet mealtime insulin dosing, which may improve compliance to prandial bolus dosing. Pharmacists in a variety of settings, including in the community and ambulatory care, can effectively provide this education to promote compliance.

Further, there was no increase in hypoglycemia with use of V-Go, rather a meaningful decrease in clinically relevant hypoglycemia < 55 mg/dL, in the overall population after a mean of 5 months of V-Go therapy. This may be beneficial to decrease hypoglycemia-associated serious adverse events and, subsequently, healthcare system financial burden [4,25]. Use of V-Go was not associated with significant changes in weight in patients previously treated with insulin, which can be common with insulin therapy [26].

### Limitations

This retrospective study did not include a control group, and therefore only associations can be made. This design was limited to the data available and is representative of a single clinic and provider. Data may not be generalizable to the broader population; however, data are consistent with other research findings for V-Go. 

Per protocol, an A1C within six months of V-Go initiation was required for a patient to be included in the study. Variation in the number of visits per patient and in the length of time between visits may have impacted the study results. In addition, prescribed insulin and NIGLM doses were extracted from the medical records but these doses may have differed from the actual doses administered. Other factors may have contributed to the reduction in A1C including the frequency of self-monitoring blood glucose levels and adherence to a modified nutrition plan or exercise regimen. Evaluation of these factors was not possible as they were not consistently captured in the medical records. However, these factors were not newly introduced upon initiation of V-Go. Finally, the presence or absence of hypoglycemia was patient-reported at clinic visits, and therefore the actual prevalence may have differed. 

## 5. Conclusions

Results of this real-world study support the clinical benefits of insulin therapy with V-Go. When patients with suboptimally-controlled diabetes switched from prior anti-hyperglycemic regimens to basal-bolus insulin therapy using V-Go, there was a statistically significant reduction in A1C as well as a significant improvement in patients achieving an A1C goal of < 8%. This was achieved while using less insulin and with a reduction in clinically relevant hypoglycemia. Incorporating such devices could be beneficial for pharmacists to consider when patients are needle adverse, struggle with adherence, or face other barriers to traditional methods of insulin delivery. Patch-like insulin delivery devices such as V-Go are an important advancement in diabetes technology that can improve care for patients and support pharmacists and other healthcare professionals in achieving clinical performance indicators. 

## Figures and Tables

**Table 1:** Baseline Patient Characteristics (N = 139).

Characteristic	N (%) or Mean ± SD
Age	59.98 ± 11.60
Sex	
Female	81 (58)
Male	58 (42)
Race	
White	115 (83)
Black	19 (14)
Asian	1 (<1)
Biracial/multiracial	1 (<1)
Not reported	3 (2)
Duration of diabetes (years)	13.82 ± 8.72
A1c (%)	9.80 ± 1.62
A1c < 8%	20 (14)
Body mass index, kg/m^2^	35.03 ± 6.10
Weight, kg	102.40 ± 18.98
Insulin daily dose (u/day)	63 ± 39
Insulin regimen	
Basal-only	73 (53)
Basal-bolus	36 (26)
Premix	13 (9)
Naïve	17 (12)

**Table 2:** Concomitant NIGLM Therapy at Baseline and Study Completion (N = 139).

NIGLM	Baseline (Pre V-Go) N (%)	Study Completion (On V-Go) N (%)
At least 1 NIGLM	121 (87)	115 (83)
Metformin	65 (47)	61 (44)
Sulfonylurea	62 (45)	37 (27)
GLP-1 agonist	28 (20)	35 (25)
DDP-4 inhibitor (DDP-4 I)	21 (15)	16 (12)
SLGT-2 inhibitor	18 (13)	25 (18)
Thiazolidinedione	13 (9)	15 (11)
DPP-4 I/metformin	9 (6)	8 ()
Metformin/sulfonylurea	2 (1)	2 (1)
Other ^1^	14 (10)	8 (6)

^1^ Bromocriptine, nateglinide, dapagliflozin/metformin, colesevelam, SGLT-2 inhibitor/metformin, and pramlintide.

**Table 3:** Hypoglycemia at Baseline and on V-Go (N = 139).

	Baseline (Pre V-Go) N (%)	Study Completion (On V-Go) N (%)	*p*-Value
Hypoglycemia < 55 mg/dL	15 (11%)	9 (6%)	0.200
Hypoglycemia < 70 mg/dL^1^	38 (27%)	34 (24%)	0.239
No hypoglycemia	98 (71%)	103 (74%)	
Not reported	3 (2%)	2 (1%)	

^1^ Inclusive of < 70 and <55 mg/dL.
